# Enema fumosum: the contribution of the North American first nations to drowning resuscitation – A historical review

**DOI:** 10.1016/j.resplu.2026.101366

**Published:** 2026-05-20

**Authors:** Ludwig Brandt, Ulrike Artmeier-Brandt, Klara Luisa Brandt

**Affiliations:** a Emeritus, Faculty of Medicine, University of Witten/Herdecke, Witten, Germany; b Specialised Ethics Committee, BfArM, Cologne, Germany; c Faculty of Medicine, Vilnius University, Vilnius, Lithuania

**Keywords:** Age of enlightenment, History, Inversion method, JAMA, Tobacco smoke, Rectal insufflation

## Abstract

Rectal insufflation of tobacco smoke was the most used resuscitation method for drowning accidents in the second half of the 18th century. Probably practised for centuries by the indigenous tribes of Canadian Acadia, knowledge of this form of treatment reached England from the New World already at the beginning of the 17th century. The East India Company’s fleet surgeon, John Woodall, was the first to describe the method in his textbook, published in its second edition in 1639, entitled “The Surgeons Mate” and referred to it as “Enema fumosum”. Within a few decades, this treatment spread throughout Europe, initially being used primarily to treat severe abdominal complaints. Then in 1740, at the behest of Louis XIV the French polymath René-Antoine Ferchault de Réaumur recommended tobacco smoke enemas as the most effective method of resuscitation for drowning victims. The rescue organisations that emerged shortly thereafter in many European countries, such as the Dutch “Maatschappij tot Redding van Drenkelingen”, founded in 1767, adopted Réaumur’s recommendation. Emergency literature of the time contains hundreds of examples of successful resuscitation after using this method. The first doubts about its effectiveness were expressed in 1776 by the famous English surgeon John Hunter, followed shortly afterwards by the physicians Charles Kite and James Curry. The method fell increasingly into disrepute. By the middle of the 19th century, it had been definitively replaced by Marshall Hall’s “Ready Method” and other new resuscitation methods. What remains of it today are memories of a form of therapy that seems curious, but whose implementation undoubtedly saved the lives of a large number of people threatened with drowning as many reports on successful drowning resuscitations demonstrate.

## Introduction

In the second half of the 17th century, the dawn of the Age of Enlightenment, London and Paris were the world capitals of science. Scholars from all disciplines – many of them polymaths – met to present and exchange ideas and new findings at the “Royal Society”, founded in 1660 in England, and the “Académie des Sciences”, founded in 1666 in France. The first regularly published scientific journals were the “Philosophical Transactions” in London from 1665 and the “Mémoires de l'Académie Royale des Sciences” in Paris from 1666.

One of these polymaths was the Frenchman René-Antoine Ferachault de Réaumur^1^ (1683–1757), still known today as the creator of the temperature scale named after him. What is not so well known is that he is also considered one of the key founders of modern resuscitation medicine, especially of lay resuscitation of drowning victims.

At the behest of Louis XIV, Réaumur published an “*AVIS pour donner du secours à ceux que l'on croit* Noyez”, in which he addressed the inhabitants of river, lake and coastal regions and urged them to provide first aid without hesitation or resentment in the event of drowning accidents. This included measures such as rolling the drowned person in a barrel, inducing vomiting, restoring normal body temperature, moving the body, applying irritating liquids and smelling salts, and antegrade (nasal/oral) or retrograde (rectal) insufflation of air. A doctor who might arrive could also perform bloodletting and a tracheotomy, through which fresh air could be blown into the drowned person.

However, Réaumur recommended that the best and most effective measure was to administer tobacco smoke to the drowned person via the rectum: “*But perhaps the best thing is to blow tobacco smoke from a pipe into the intestines; one of our Academicians witnessed the prompt and happy effect of this smoke on a drowned person: a broken pipe can provide the tube or blowpipe through which the smoke drawn from the whole pipe can be blown into the body*” (translated from the original French text).

This so-called “tobacco smoke enema” became so popular in the following years that the “Maatschappij tot Redding van Drenkelingen” (Society for the Rescue of Drowned Persons), founded in Amsterdam in 1767 as the world's first rescue organisation, recommended the method as the decisive measure for resuscitating drowned persons:

“*The best means that can and should be used to restore a drowned person … are as follows: Firstly, blowing into the buttocks (rectum) by means of a tobacco pipe or other pipe, or a knife sheath, the extreme tip of which is cut off, or by means of a bellows itself, and the more rapidly, strongly, and persistently this blowing is performed, the much better it will always be. … This blowing, however, must be the very first action, in one or other of the ways indicated*”.^2^

## Where did the idea come from to blow tobacco smoke into the buttocks of drowned people to revive them?

The treatment of a wide variety of ailments with liquid, steam or smoke enemas has been practised for thousands of years by almost all peoples and cultures. The ancient Egyptians, for example, worshipped the sacred bird Ibis as the inventor of the enema. And at the end of the 17th century, German professor of medicine Johann Andreas Stisser wrote that enema instruments were “a *Barbaris aut Turcis* inventa”.^3^

Although the enema technique had been known for a variety of indications the special method of tobacco smoke enema for drowning resuscitation only emerged in the 17th century, when the idea of “First Aid” came up and when tobacco had already been recommended as a “Panaceum” in Europe.

Like corn, potatoes and tomatoes, tobacco is one of the genuine American plants that only reached Europe and the rest of the world after Columbus' discovery in 1492. The Spanish and Portuguese brought the new plants home from Central and South America, while the English and French brought them home from North America.^4^ Sailors such as Walter Raleigh and Francis Drake introduced tobacco to England, while Jacques Cartier and Samuel de Champlain did so in France. On the Iberian Peninsula, it was, among others, the monk Ramon Pané, who accompanied Columbus on his second voyage to America, the Dominican Bartolome de las Casas, Fernández de Oviedo y Valdes and Nicolás Monardes. Oviedo y Valdes was, incidentally, the first to use the name “tobacco” (“tabaco”).^4^

Although most of the authors mentioned report on American First Nations’ medicine and on different uses of tobacco, none of them referred to tobacco smoke enemas. Nevertheless, it stands to reason that the method was imported from the New World. This is supported, on the one hand, by the fact that the tobacco plant was exclusively native to America and known there until Columbus’ discovery. On the other hand, the method is attributed to the First Nations in two reliable sources, although these were published more than two hundred years later.

In 1744, the Frenchman Pierre François Xavier de Charlevoix^5^ published his “*Histoire et description générale de la Nouvelle France*”. In the section on the year 1611, he recounts the following story, citing the lawyer and traveller Marc Lescarbot^6^ and the Jesuit Pierre Biard^7^: “*These savages had a rather peculiar way of reviving those who were in danger of drowning and had swallowed a lot of water. They filled an animal bladder or a large, wide intestine, which was tightly tied at one end, with tobacco smoke; they attached a cannula to the other end and inserted it into the patient's anus; then they squeezed the intestine or bladder to direct the smoke into his body. Then they hung him by his feet from a tree, and the smoke that filled his stomach caused him to expel all the water he had swallowed through his mouth*” (translated from the original French text).

However, neither in Lescarbot's “*Histoire de la* Nouvelle-France”, published in 1609, nor in Biard's “*Relation de la nouvelle France*”, published in 1616, is there a passage that would indicate this “custom” of the Canadian First Nations. So, from which source did Charlevoix quote?

The answer can be found in the 1708 publication “*Relation du Voyage du Port Royal de l'Acadie, ou de la nouvelle France*” by the French physician and botanist Marin de Diereville.^8^ He reports the following: “*Let us return to the savages who heal themselves from death itself; what a paradox, one might say! But I can prove it. These poor people are prone to drowning, and this happens all too often in their bark canoes, which capsize at the slightest disturbance. Those who luckily escape the shipwreck rush to pull those who remain in the water out; they fill an animal stomach or a large, long intestine, their usual vessels for storing fish or seal oil, with tobacco smoke; after that, they attach a piece of calumet or pipe to one end, the other end being securely tied, to serve as a cannula, which they insert into the rear of the drowned, to make them receive the smoke contained in the intestine, compressing it with their hands: They then hang them by their feet from the nearest tree they can find, where they observe them, and they almost always have the pleasure of seeing that this vapour enema causes them to expel all the water they have taken in and restores life to their bodies. They recognise this surprising and salutary effect by the wriggling movements that the hanged men soon begin to make. Do not forget this divine remedy, proven by thousands of experiments, its virtue in your friends, same as in savages*” (translated from the original French text).

It was therefore the indigenous people of historical Acadia, the Mi'kmaq, Maliseet and Abenaki tribes, who first introduced European sailors to tobacco smoke enemas as a method of resuscitation. Historical Acadia roughly covered the area of the present-day Canadian provinces of Nova Scotia, New Brunswick, Prince Edward Island and, with the southern part of the Gaspé Peninsula, also parts of the province of Québec. It also included the north-eastern part of the US state of Maine.

## When and how did tobacco smoke enemas come to Europe?

As already mentioned, tobacco was introduced to Europe by sailors and travelling scientists as a stimulant, but also as a therapeutic agent. People were so convinced of its therapeutic effects that it was soon touted as a “panacea”, a cure-all. There were a wide variety of indications and preparations for its use, either transdermal, as a decoction for oral use, as smoke for inhalation, or even for rectal application.

**Fig. 1:**
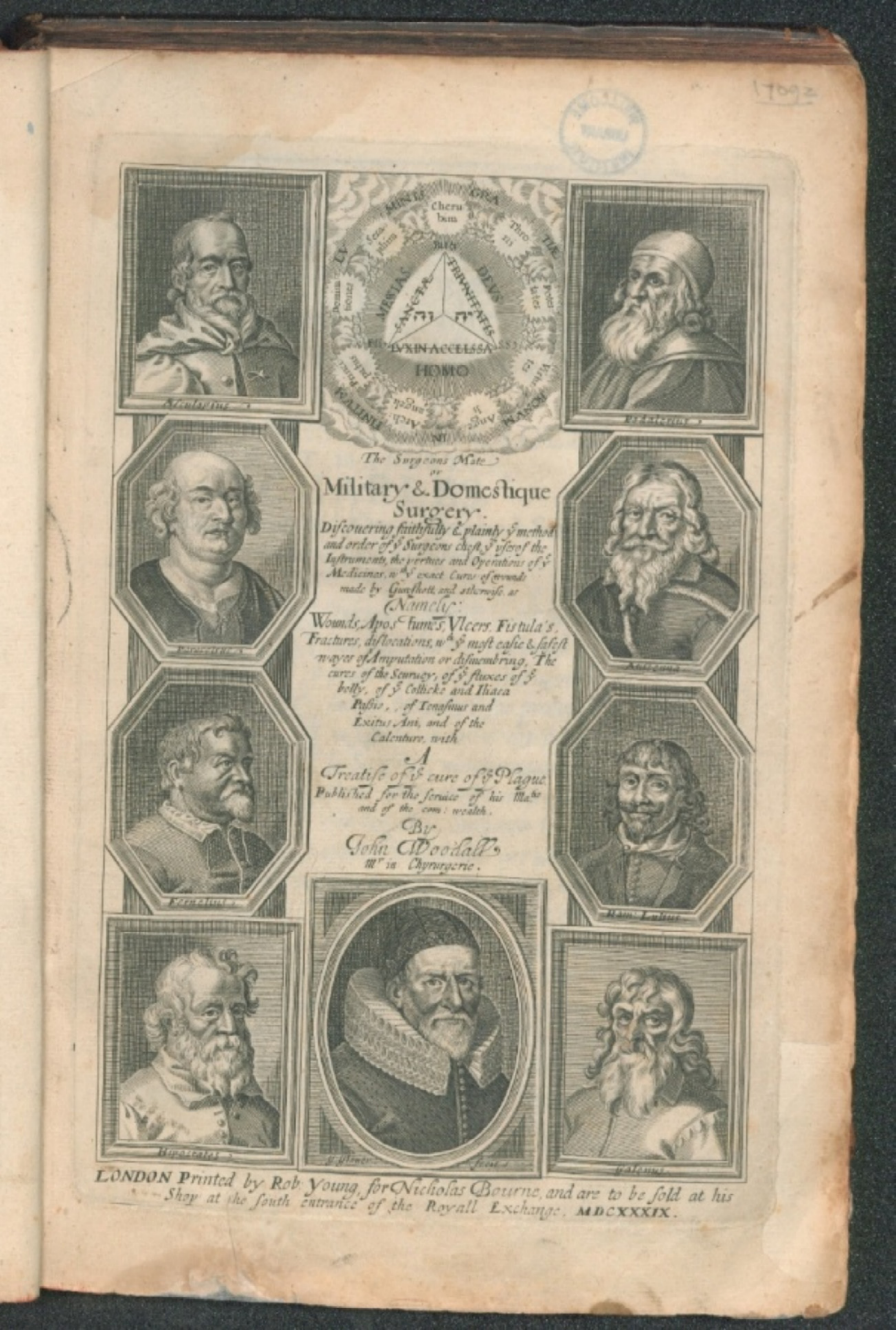
**Titlepage of the 2nd Edition of John Woodall’s “The Surgeons Mate”, 1639 (Public Domain)**.

**Fig. 2:**
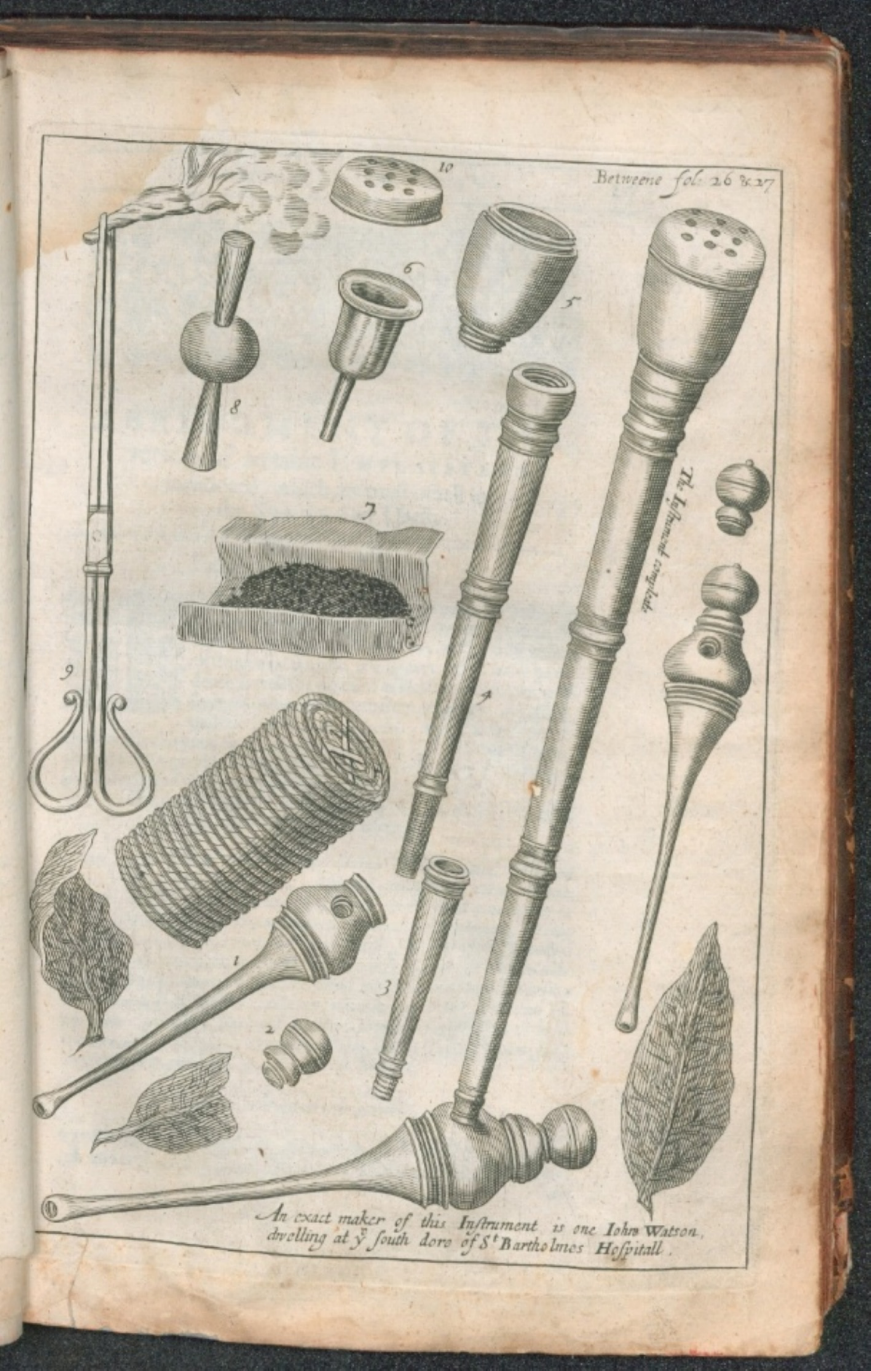
**Woodall’s instruments for the enema fumosum (public domain)**.

In 1639, the English military surgeon John Woodall^9^ published the second edition of what was then the standard medical text for naval doctors, “The Surgeons Mate” (cf. Fig. 1). The book contains a text of just under five pages and an illustration of an “Enema fumosum or a fumous glister” (cf. Fig. 2):

“*Being a new found Art of giving a Glister of smoke wind, any dry powder whatsoever in substance, into any man's body, very convenient in many occasions, experimented, to be not only safe; but also most comfortable, pleasant, profitable, and easy to be perceived in; being a most profitable instrument, and art for the way of curing many grievous infirmities, which although in all occasions either domestic or military, it may be very useful and good; yet, it may be esteemed most necessary and expedient for the military surgeon*”.

**Fig. 3:**
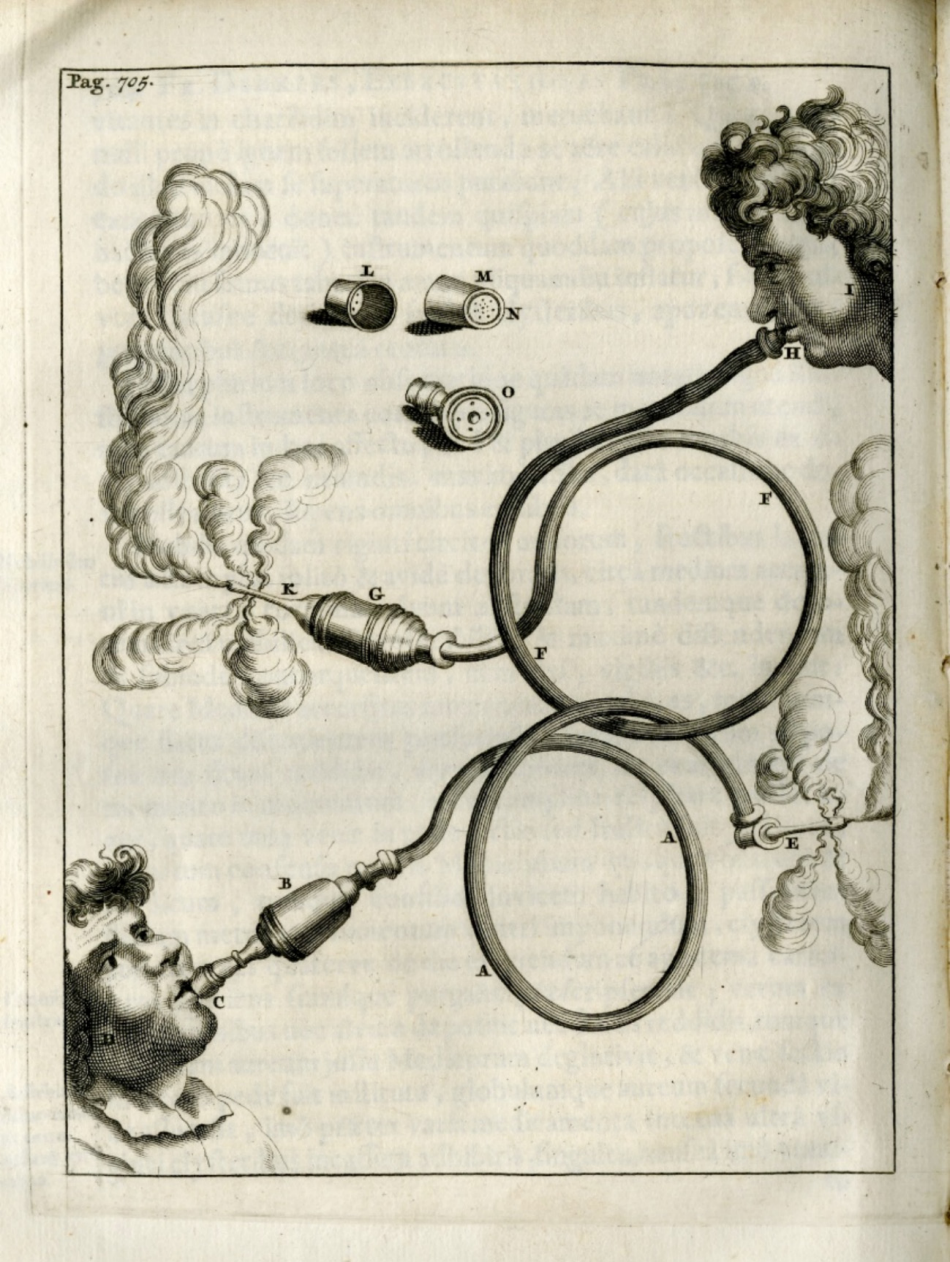
**Table from Frederic Dekker’s “Exercitationes practicae” 1694 (©L. Brandt)**.

Woodall's description had a massive impact. The tobacco smoke enema of the Canadian natives had not only arrived in England but also spread like wildfire throughout the rest of Europe. And hardly any of the standard medical works of the later 17th century failed to mention it because of its excellent reputation, especially for grievous abdominal problems. For example, Thomas Bartholinus^10^ mentioned an “*instrumentum conficiunt ingeniosi Angli*”, Regnier de Graaf^11^ described a “*funiculus quem Angli ad fumum tabaci in anum inflandum introduxerunt*”, Johann Andreas Stisser^12^ mentioned an “*Instrumentum anglicanum in fundo habens*”, and Lorenz Heister^13^ wrote, “*In England, they have also devised a method of blowing tobacco smoke into the anus using special instruments*”. One of the most beautiful illustrations of such an instrument can be found in Frederic Dekker’s^14^ work (cf. Fig. 3).

The central theme of emergency medicine in the 17th and 18th centuries was drowning accidents. And so, it was only a matter of time before the enema fumosum was also recommended for this indication. The first recommendations for laypeople on resuscitating drowning victims appeared as early as 1620,^15^ but scientific and public interest in the subject was only aroused by a series of articles published in the monthly magazine “*Mercure Suisse*” between 1733 and 1735. Réaumur also referred to these in his Avis. Among the first aid measures propagated for drowning accidents, the idea of resuscitating drowned persons by rectal air insufflation was not new in the 1740s. The English clergyman and chaplain of the Royal Navy, Henry Teonge,^16^ recorded the following incident in his diary already on 12 June 1675:

“Fair weather on Saturday. But so tempestuous on Sunday that many said they had never seen such weather there at that time of the year. This day at Deale Beach a boat was overturned with five men in it: three leapt out and swam to shore with much difficulty; the other two were covered by the boat, one of whom was dead and sank; the other, whose name was Thomas Boules, (when the boat was pulled off him, which had lain on his head and neck a long time,) was carried away with the violence of the water; yet in sight, and by that means was at last hauled out, and there lay on the stones for dead; for his fellow was dead long before. A traveller, in very poor clothes, (coming to look on, as many more did,) presently pulled out his knife and sheath, cut off the lower end of his sheath, and thrust his sheath into the ***** of the said Thomas Boules, and blew with all his force till he himself was weary; then desired some others to blow also; and in halfe an howers time brought him to life again. I drank with him at his house, 28 April 1678”.

**Fig. 4:**
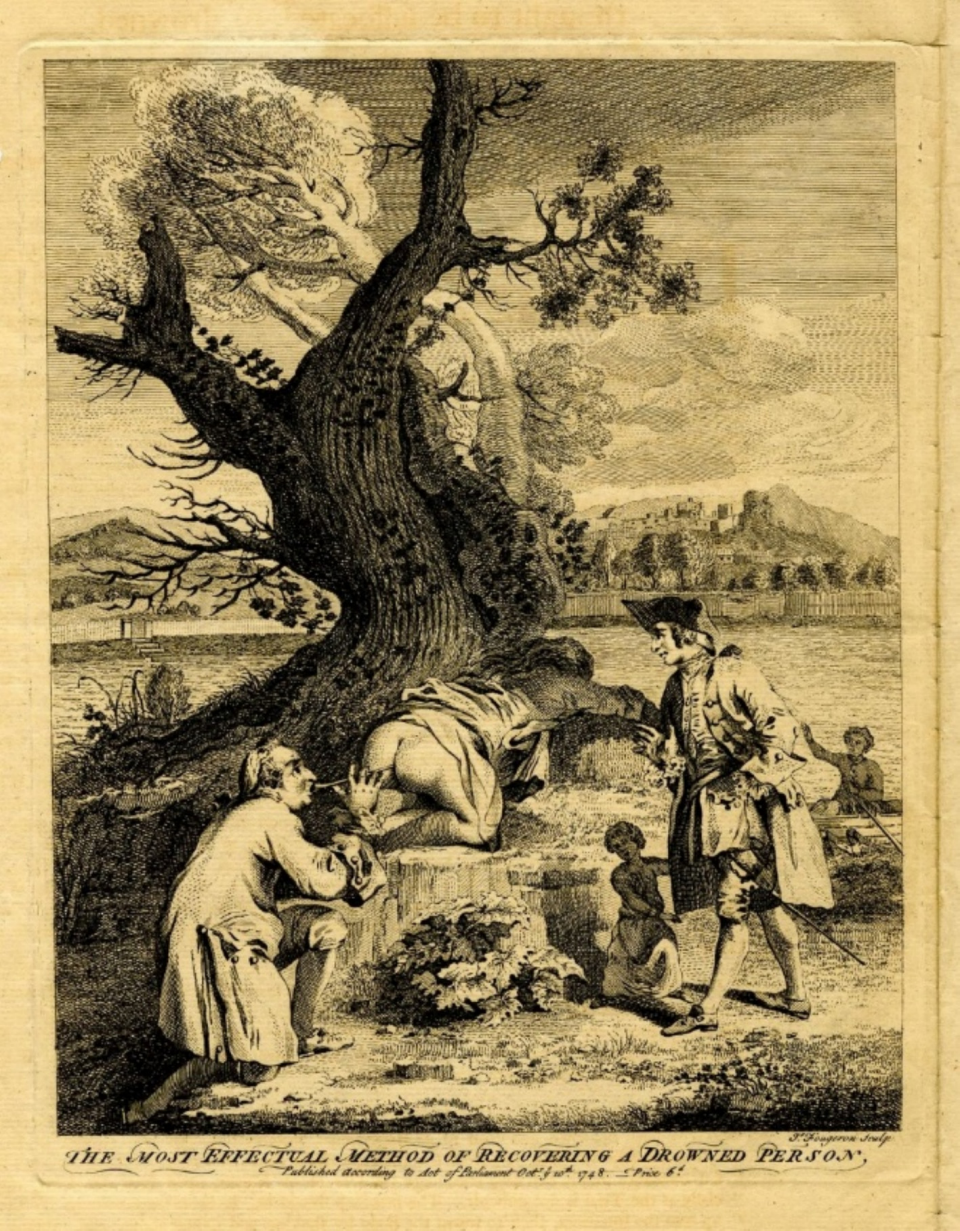
**Pamphlet 1747 (unknown author): The most effectual method of recovering a drowned person. The British Museum (public domain)**.

The measure of blowing air into the anus of a drowned person to resuscitate them was, therefore, apparently already known in Europe and was used alongside other measures. It may be that word of the method had already spread among sailors from the New World, and that the “traveller in very poor clothes” only came up with the idea of using a knife sheath because he did not have a pipe. However, it remains to be noted that Réaumur only had to take a small step to establish the First Nations' resuscitation method in Europe. For what could be more obvious than to use this panacea from the New World in such life-threatening situations? Réaumur was helped in this by an incident reported to him by one of the members of the Académie^17^, ^18^:

“*Mr Thomas, a surgeon in Paris, living in Passy, was waiting in a boat for the carriage to be full. During this time, he approached someone who had just crossed the river. He picked up a man who, disembarking with the other passengers, asked what had become of his wife, but no one could give him an answer except a very young child, as will be seen below, who, pointing to the river, told him that she had hidden there. She had fallen from the stern of the boat where she had been holding on during the crossing, without anyone but the child noticing. The husband immediately jumped into the boat, with only the child to guide him, and turned back to find his wife in a shallow but muddy spot. She was pulled out and brought to the shore, where she was laid down. While some advised hanging her by her feet and others suggested other methods, a soldier passing by with a pipe in his mouth approached to find out what had gathered such a crowd. Informed of the situation, he told the husband to dry his tears, and that his wife would soon be alive again. Then, giving his pipe to the husband, he told him to insert the stem into her anus and blow smoke into it with all his might, placing the bowl covered with paper pierced with several holes in his mouth. At the fifth puff of smoke, a considerable stirring was heard in the woman's belly; she vomited water from her mouth, and a moment later she regained consciousness. She was placed on her feet. However, as Mr. Thomas's boat was now full, he set off after assuring the husband that his wife's life was safe. He turned his head several times to see how the adventure would unfold, and finally saw the woman being taken to a nearby tavern, where she regained the strength necessary to return home*” (translated from the original French text; cf. Fig. 4).

Here, too, it seems reasonable to assume that the tobacco smoke enema used by the North American Natives had long been known in certain circles, e.g. among sailors and soldiers, before it was recognised by science. And something else seems interesting in this context: In completely different cultures, which had no contact with each other before Columbus' discovery, the same method was used among others in attempts to resuscitate drowned people: Because it was assumed that the drowned person had swallowed water, one of the first measures taken was to hang them by their feet so that they would regurgitate the water they had swallowed. This was already the practise among the ancient Egyptians almost 3500 years ago, as well as among the Greeks and Romans.^19^

## The fate of the enema fumosum

**Fig. 5:**
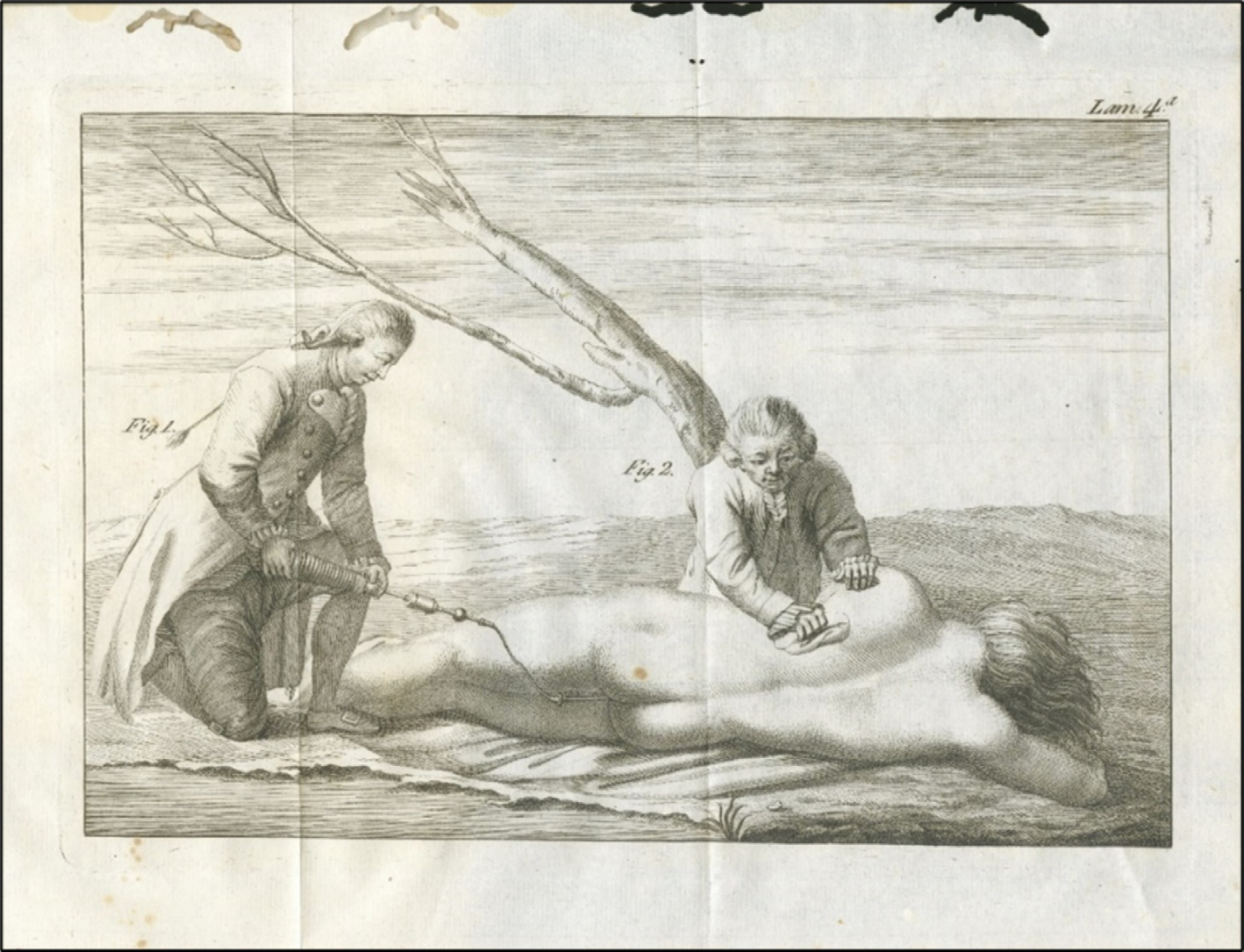
**Resuscitation of a drowned person 1784 (©L. Brandt)**.

For more than 50 years, the tobacco smoke enema remained the method of choice for resuscitating drowned persons in Europe. In countless scientific and literary publications, it was recommended as the superior measure to all others (cf. Fig. 5).^20^

John Hunter^21^ (1728–1793), one of the most famous English surgeons, was the first to doubt the effectiveness of this form of treatment when he wrote in the Philosophical Transactions in 1776: “*I would likewise avoid throwing anything in by the anus, which might tend to an evacuation that way, as every such evacuation also tends to lessen the animal powers. Of course, I have avoided speaking of the fumes of tobacco, which always produce sickness or purging, according as they are supplied*”*.* But Hunter was not yet ready to completely abandon the enema fumosum. Among the first-aid instruments he promoted, in addition to a bellows for nasal or oral resuscitation, there was still, “*a pair of small bellows, such as are commonly used in throwing fumes of tobacco up the anus*”*.*

Ultimately, it was the Englishmen Charles Kite^22^ and James Curry^23^ who attributed more harm than good to the method. Kite wrote: “*Considering all circumstances, then, is it not a just inference, that although tobacco may at first act as a stimulus, yet it will afterwards, by its narcotic and deleterious properties, not only counteract what it has accomplished, but will abolish what before existed*”*.*

Curry took an even clearer stance just four years later: “The intestines, due to their internal location and peculiar constitution, retain their irritability longer than other parts of the body, and, accordingly, various means have been proposed for increasing the action of their fibres in order to restore the activity of the entire system. Tobacco smoke, injected by way of glyster, is what has been generally employed with this view, and the fumigator, or instrument for administering it, forms part of the apparatus, which is currently distributed by the various societies established for the recovery of drowned persons. Of late, however, the use of tobacco smoke has been objected to, and upon very strong grounds; for when we consider that the same remedy is successfully employed with the very opposite intention, namely, that of lessening the power of contraction in the muscles, and occasioning the greatest relaxation consistent with life, it must be acknowledged to be a very doubtful, if not dangerous remedy, where the powers of life are already nearly exhausted”.

**Fig. 6:**
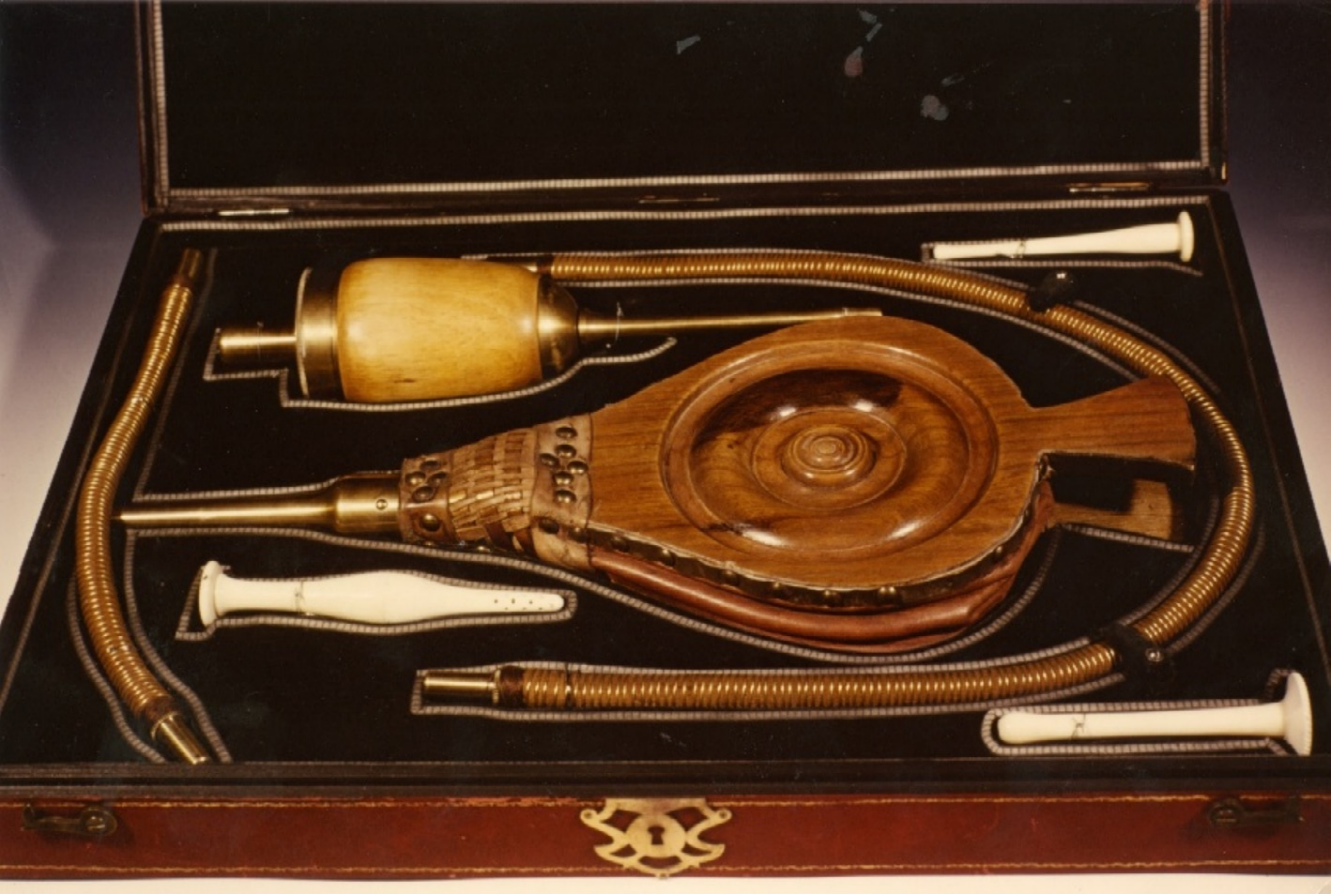
**“Fumigation Set” for rectal application of tobacco smoke; Austrian Tobacco Museum Vienna (©L. Brandt)**.

At the beginning of the 19th century, the London Royal Humane Society finally rejected tobacco smoke enemas as a useful resuscitation measure. Nevertheless, the method – and the instruments designed for this purpose, some of which were very elaborately crafted (cf. Fig. 6)^24^ – remained part of the standard resuscitation repertoire for many more years.^25^ Finally, towards the end of the 19th century, the contribution of the North American Natives gradually disappeared completely from the emergency medical repertoire. It was increasingly recognised that adequate oxygen supply through appropriate forms of artificial respiration, electrical stimulation of the heart and the intravenous administration of drugs were the more promising measures for successful resuscitation. As early as 1776, John Hunter had written in his “Proposals for the Recovery of People apparently drowned”*:* “…*blowing air into the lungs may be sufficient to effect a recovery*”*.* And further: “*How far electricity may be of service, I know not; but it may, however, be tried, when every other method has failed*”*.*

When Marshall-Halls' “Postural Method” was finally introduced in 1857, ushering in the era of artificial respiration without additional instruments, meaning that resuscitation could always be started immediately with a “Ready Method”, the tobacco smoke enema finally became a thing of the past. Thus, a method that had been considered proven for many decades and praised by numerous contemporary witnesses came to an end.^19^, ^24^, ^25^

## What memory of the first Nations' contribution remains?

**Fig. 7:**
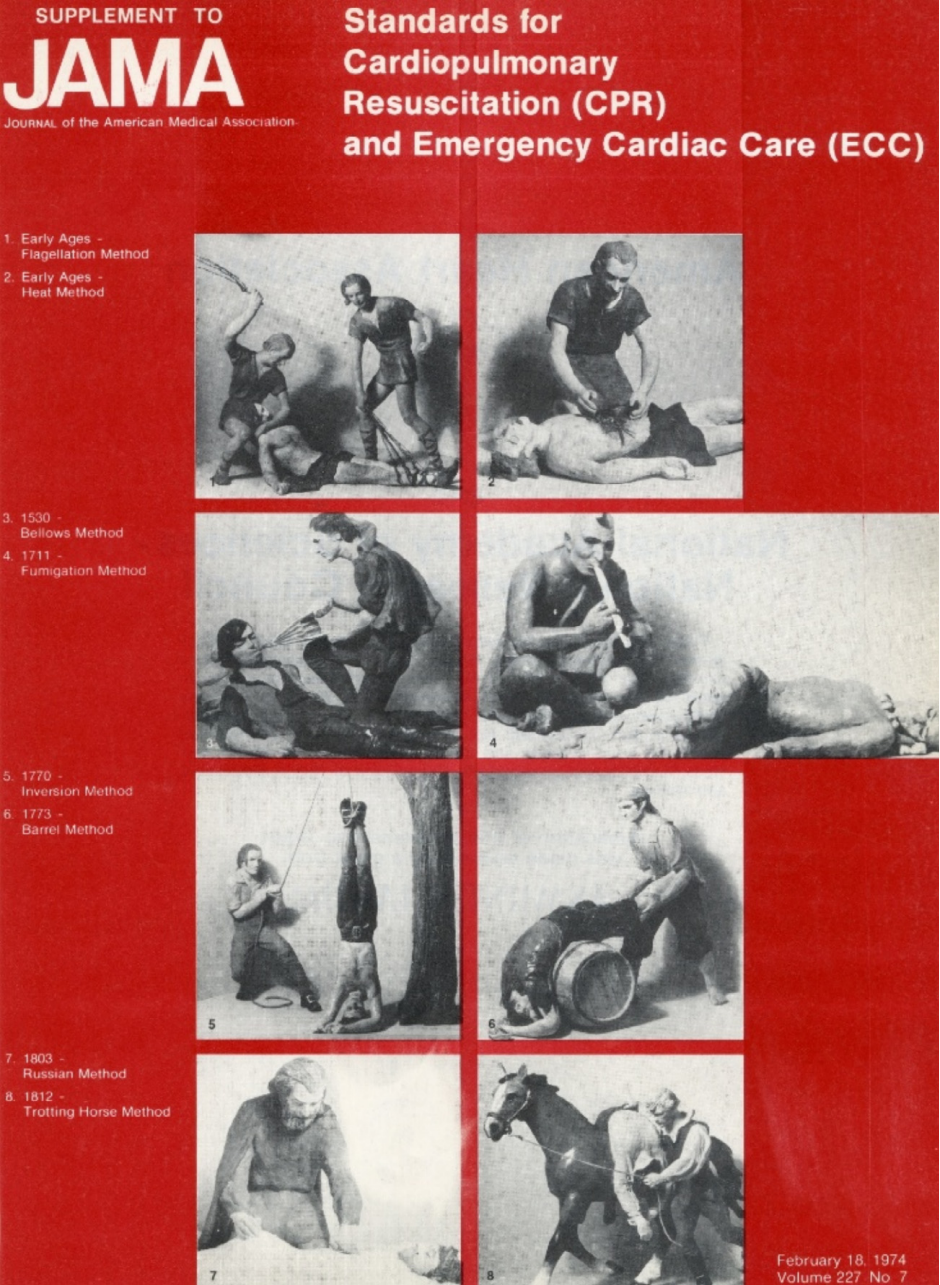
**Title page JAMA 1974;227 Supplement**.

When the American Heart Association first published its “Standards for Cardiopulmonary Resuscitation (CPR) and Emergency Cardiac Care (ECC)” in a supplement to the Journal of the American Medical Association (JAMA) in February 1974,^26^ it used as “cover illustrations” several methods of historical resuscitation measures that originated from an earlier exhibition at the Museum of Science and Industry in Chicago (cf. Fig. 7). There, the scenes, originally created as three-dimensional wax models, had fallen victim to flames in 1963 and are now only preserved in photographs.

Among the methods, most of which are now considered curious, is the “*Fumigation* Method” of the First Nations, presented as method no. 4. The explanatory text accompanying the illustration reads: “*North American Indians attempted to revive apparently dead persons by blowing smoke into an animal bladder and therefore into the victim's rectum. Also called 'Dutch fumigation', it was introduced into England in 1767. The method was used successfully for years in American colonies*.” At least, even if the historical context was not represented entirely accurately, the contribution of the North American First Nations is not completely forgotten.

Today, we may smile at the First Nations' enema fumosum and doubt whether it was as effective as was assumed. However, given the documented success stories, there can be no doubt that the method saved hundreds, perhaps even thousands of lives from drowning,^2^, ^17^ even if the underlying pathophysiology may seem cryptic according to today’s understanding.^19^, ^24^ Basically, it doesn't matter whether it was the Enema fumosum that led to a successful resuscitation. What was much more important was that efforts were made to save the victims from drowning.^21^, ^25^

## Confirmation

The manuscript, including related data, figures and tables, has not been published previously and is not under consideration elsewhere.

## CRediT authorship contribution statement

**Ludwig Brandt:** Supervision, Resources, Conceptualization. **Ulrike Artmeier-Brandt:** Writing – original draft, Validation, Project administration. **Klara Luisa Brandt:** Writing – original draft, Visualization, Investigation.

## Declaration of competing interest

None.
